# Skin Grafting

**Published:** 1899-09-30

**Authors:** 


### SKIN GRAFTING.

From small beginnings, the size of the pieces of skin employed in skin grafting has gradually increased, and now for some years fashion seems to have decided that the large sheets used in the Thiersch graft is the best for most purposes. As a sort of rebound from this extreme we find ourselves now introduced to a method, that of Dr. Wiggins, described in the *St. Louis Clinique*, which aims at sowing sterile epidermic scales over the surface of the wound. Of course epidermic scale grafting has been done in a rough and ready manner many times before, but in the new method the details appear to have been carefully arranged with the object of attaining the desired end. The epidermis is derived from the sole of the foot, where its growth is specially luxuriant. The sole is first thoroughly scrubbed so as to remove as far as possible the outer and germ-laden layer of epidermis. It is then bathed in strong perchloride solution, after which it is covered with moist boracic acid dressing and rubber tissue, and after this has been on for twelve hours, the dressing is removed and the surface thoroughly scraped with a dull knife. The mass of epidermis thus obtained is placed in a mortar heated to 110 deg. to 115 deg. Fahr. by a water bath, and stirred until thoroughly desiccated. By this means not only is the mass broken up into a fine powder, but the fragments become so dry that they adhere at once to surface of the ulcer when applied to it. This epidermial powder is then sown over the whole of the granulating ulcer to be treated, which if it be a small one, will sometimes be found to be covered with a thin film of organised tissue within 24 hours. If the ulcer is two inches wide or upwards certain other proceedings will be desirable to prevent the scales being washed away by the discharge, or lifted off the ulcer by exudate below them. Either the scales may be applied only to a strip a quarter of an inch wide round the edge of the ulcer, and then covered with rubber tissue, the rest of the ulcer being dressed with an absorbent dressing, or a piece of rubber tissue with small perforation may be stretched all over the ulcer, and the epidermic scales may then be sown on to the protruding granulations. The results are said to be very good.

